# Harnessing the Genetic Plasticity of Porcine Circovirus Type 2 to Target Suicidal Replication

**DOI:** 10.3390/v13091676

**Published:** 2021-08-24

**Authors:** Agm Rakibuzzaman, Pablo Piñeyro, Angela Pillatzki, Sheela Ramamoorthy

**Affiliations:** 1 Department of Microbiological Sciences, North Dakota State University, Fargo, ND 58102, USA; agm.rakibuzzaman@ndsu.edu; 2 College of Veterinary Medicine, Iowa State University, Ames, IA 50010, USA; pablop@iastate.edu; 3 Animal Disease Research and Diagnostic Laboratory, South Dakota State University, Brookings, SD 57007, USA; angela.pillatzki@sdstate.edu

**Keywords:** porcine, circoviruses, PCV2, immunity, cell-mediated immunity, innate immunity, antibody, vaccine, evolution, codon, mutation, immunopathogenesis

## Abstract

Porcine circovirus type 2 (PCV2), the causative agent of a wasting disease in weanling piglets, has periodically evolved into several new subtypes since its discovery, indicating that the efficacy of current vaccines can be improved. Although a DNA virus, the mutation rates of PCV2 resemble RNA viruses. The hypothesis that recoding of selected serine and leucine codons in the PCV2b capsid gene could result in stop codons due to mutations occurring during viral replication and thus result in rapid attenuation was tested. Vaccination of weanling pigs with the suicidal vaccine constructs elicited strong virus-neutralizing antibody responses. Vaccination prevented lesions, body-weight loss, and viral replication on challenge with a heterologous PCV2d strain. The suicidal PCV2 vaccine construct was not detectable in the sera of vaccinated pigs at 14 days post-vaccination, indicating that the attenuated vaccine was very safe. Exposure of the modified virus to immune selection pressure with sub-neutralizing levels of antibodies resulted in 5 of the 22 target codons mutating to a stop signal. Thus, the described approach for the rapid attenuation of PCV2 was both effective and safe. It can be readily adapted to newly emerging viruses with high mutation rates to meet the current need for improved platforms for rapid-response vaccines.

## 1. Introduction

In the last few decades, the number of newly emerging or re-emerging viral infections has increased significantly. Over 80% of these emerging or re-emerging infections are caused by RNA viruses. The highly mutable nature of pathogenic RNA viruses and the associated antigenic and genetic diversity is a long-standing challenge for effective vaccine development [1,2]. While much attention has been focused on approaches for developing rapid attenuation methods for RNA viruses, ssDNA viruses, such as porcine circovirus type 2 (PCV2), also have a high frequency of mutation. In general, attenuated vaccines are more effective and provide a longer duration of immunity than inactivated vaccines. However, achieving the optimal balance between safety and efficacy is a challenge in developing attenuated vaccines [1]. The long lead development time associated with conventional methods of viral attenuation and the knowledge of well-characterized virulence determinants being a pre-requisite for rational reverse genetics-based approaches hinder rapid-response vaccine development for emerging viruses [1,2]. Therefore, the availability of broadly applicable vaccine platforms that are safe and effective is a critical need. We have previously demonstrated that a minimally replicative vaccine against an enteric swine coronavirus [3] is highly effective at priming the immune system but is also quickly eliminated from the vaccinated host. Thus, minimally replicative vaccines can provide a much-desired amalgamation of vaccine safety and efficacy properties.

Porcine circovirus type 2 (PCV2) is a small, circular, single-stranded DNA virus that emerged in the mid-1990s as the cause of post-weaning multisystemic wasting disease syndrome (PMWS) in weanling pigs. Thereafter, several other clinical manifestations involving the respiratory, enteric, reproductive, and immune systems were attributed to PCV2 and collectively named porcine circovirus-associated diseases (PCVAD) [4]. Currently available commercial vaccines against PCV2 consist of inactivated and subunit vaccines that target the first detected PCV2a subtype and were introduced in the U.S. in 2006. They are highly effective in controlling clinical PCVAD and reducing the economic impacts of PCVAD but do not prevent infection or viral transmission [5,6]. The selection and emergence of variants that adapt to pre-existing immunity induced by either prior infection or vaccination is a well-documented phenomenon of several viruses, including PCV2 [6,7,8], canine parvovirus [9], hepatitis B virus [10], Japanese encephalitis virus [11], and Marek’s disease virus [12]. Similarly, since the discovery of PCV2, periodical shifts in the circulating subtypes have occurred, with the first detected PCV2a subtype being replaced by PCV2b, and more recently by the PCV2d subtype, which is now the predominating field strain [13], suggesting that the exploration of alternative approaches to vaccination is warranted [8]. Current PCV2 vaccines contain either the recombinant subunit PCV2a capsid protein or the whole inactivated virus and are extremely safe. However, live attenuated vaccine approaches can potentially improve the breadth and threshold of protection elicited by currently available vaccines and help to eradicate PCV2 in the long term [5] but are traditionally not favored due to safety concerns such as reversion to virulence or recombination with field strains. In addition, PCV2 vaccines with built-in capabilities for monitoring vaccine compliance in the field are unavailable but have an added advantage for producers as vaccine coverage can be monitored, and herd immunity targeted effectively. Thus, the objective of this study was to develop a rapid attenuation strategy targeting suicidal viral replication for PCV2, with the goal of achieving high vaccine safety and efficacy margins; and to enable serological monitoring of PCV2 vaccine compliance by the expression of a foreign epitope tag.

While the genetic instability of RNA viruses is a well-recognized problem for effective prevention, in general, DNA viruses are considered to be more genetically stable. However, single-stranded (ssDNA) DNA viruses, especially the circular rep-encoding single-stranded DNA viruses (CRESS viruses), including PCV2 [14], have rates of mutation that range from 10^−3^ to 10^−6^ substitutions/site/year, which are comparable to RNA viruses. With the availability of metagenomic sequencing technology, two newly emerging PCV types, PCV3 and PCV4, were recently detected in clinical cases of reproductive failure, systemic inflammation, myocarditis, and porcine dermatitis and nephropathy syndrome (PDNS) [15]. Thus, ssDNA viruses are genetically diverse, can have a broad host range [16,17], and potentially adapt to new hosts following mutational events [18].

Previously established methods for viral attenuation involving recoding the viral genes include codon deoptimization, codon pair deoptimization, and introduction of a CpG/UpA dinucleotide bias. These methods destabilize the protein translation process to reduce viral fitness while retaining antigenicity through the preservation of amino acid sequence [19]. However, they involve a significant alteration to the viral genetic composition and dinucleotide composition and can potentially alter the innate immune responses that are triggered by the sensing of viral nucleic acids [20]. As serine and leucine have the highest codon redundancy among the amino acids, they can be recoded to increase the chances of accumulating stop mutations during viral replication in viruses with high mutation rates. This approach involves minimal changes to the viral genetic composition and was previously found to rapidly attenuate influenza and coxsackie viruses in vitro and in vivo [21]. An objective of this study was to assess whether the approach of destabilizing serine and leucine codons could be applied to DNA viruses with high mutation rates, using PCV2 as a model. The exact mechanisms by which ssDNA viruses achieve such high mutation rates are as yet unknown but may involve the use of atypical, low fidelity polymerases for replication and repair. Selection pressure induced by factors such as the serial passage in non-specific hosts [18] or conditions of nucleotide depletion [22,23] can influence mutation rates for single-stranded viruses. In the case of PCV2, vaccination in the field is often undertaken in seropositive piglets due to maternal antibodies or in sows with a history of previous vaccination. Hence, a secondary objective was to study the effect of immune pressure induced by sub-neutralizing antibody levels on the codon destabilized PCV2 vaccine construct. Thus, in this study, we demonstrate for the first time that recoding of serine and leucine codons can result in the rapid attenuation and suicidal replication of PCV2 (referred to as sPCV2-Vac), thus providing for an optimal blend of vaccine efficacy and safety.

## 2. Materials and Methods

### 2.1. Virus Culture and Cloning of the sPCV2 Vaccine Construct

**Figure 1:**
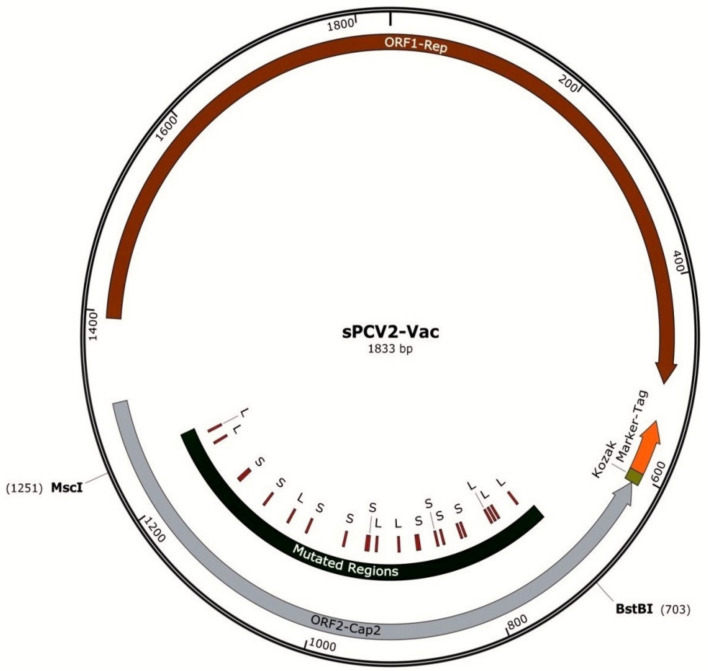
Map of the sPCV2-Vac construct: The PCV2b backbone is depicted as a circle, with the two major open reading frames indicated by counterclockwise block arrows. The transcriptional unit for the SRS2 epitope tag is shown at the 5′ end of ORF2, which encodes the capsid protein. The location of the modified serine and leucine codons is indicated and flanked by the enzymes used for insertion into the ORF2.

A previously described PCV2b infectious clone (accession KR816332) [13] was used as the backbone to develop the sPCV2b vaccine. Briefly, the PCV2b genome was amplified from the lung and liver tissues of piglets with clinical signs, using overlapping primers containing a single cutting Sac II enzyme site within the viral genome [13]. To enable the serological monitoring of vaccine compliance in the field, a foreign epitope tag was inserted in the 3′ end of the open reading frame 2 (ORF2), encoding the capsid protein as an independent transcriptional unit. As previously described [24], the epitope tag was derived from an immunogenic peptide located in the surface antigen-1 related sequence 2 (SRS2) protein (AAD04844.1) of *Neospora caninum*, an Apicomplexan parasite that does not infect pigs. Therefore, antibodies generated against the tag would be detected only in vaccinated pigs. To destabilize the serine and leucine codons, the ORF2 gene sequence between 57 and 609 bps, flanked by BstB1 to MscI enzymes, was commercially synthesized. All serine and leucine codons within the selected sequence were modified as listed in Table 1 to increase the probability that mutations at the 2nd or 3rd positions of the codon that are accrued during vaccine viral replication in the host could result in a stop codon and suicidal replication (Figure 1, Table 1). The synthesized fragment was shuttled into the infectious clone by restriction digestion. The construct is referred to as sPCV2b-Vac in the manuscript.

All recombinant virus stocks used in this study were prepared by the transfection of the PCV1 free PK 15 cell line (005-TDV, National Veterinary Services Laboratory, Ames, IA, USA). The challenge virus was generated from an infectious clone of a heterologous PCV2d strain (accession number JX535296.1) [25]. The other PCV2 strains used in the virus neutralization assays were generated from transfection of PK-15 cells with infectious clones for PCV2b (EU340258.1) and PCV2a (AF264042.1) [26]. Virus stocks were stored at −80 °C until use.

### 2.2. Rescue of Recombinant Virus Stocks

**Figure 2:**
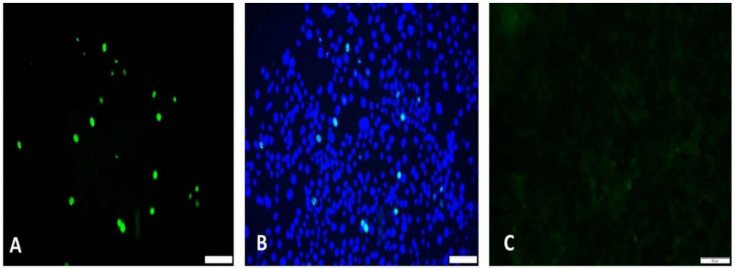
Detection of sPCV2-Vac in transfected cells: Rescue of the sPCV2 in PK-15 cells as detected by an immunofluorescence assay with PCV2-specific polyclonal antibody. (**A**)—Apple green nuclear fluorescence indicates PCV2 replication, (**B**)—Overlay image with DAPI stained nuclei in blue. (**C**)—Uninfected cell control. Bar—50 µM scale.

Virus cultures were prepared essentially as previously described with some modification [27,28]. Briefly, the PCV1 free PK-15 cell line was seeded to obtain approximately 50% confluence in T25 flasks. The PCV2 genome was excised from the plasmid vector by digestion with Sac II and circularized by ligation for the sPCV2-Vac construct. Dimerized infectious clones were used for the other viral strains. The T25 flasks were transfected with 12 µg of DNA, using the Trans IT-2020 (Mirus Bio, Madison, WI, USA) reagent, following the manufacturer’s instructions. The virus cultures were harvested by freezing and thawing the flasks after 72 h of incubation at 37 °C in a CO_2_ incubator. Virus titers were obtained in duplicate by the tissue culture infective dose (TCID_50_) method. As PCV2 does not produce visible cytopathic effects, viral replication was visualized by an immunofluorescence assay [29]. Eight well chamber slides were seeded with PK-15 cells to reach 50% confluence. To obtain the TCID_50_ values, serial log dilutions of the stock virus culture were used to infect the monolayers in 4 replicates. To visualize the rescue and replication of sPCV2-Vac, slides were transfected with the construct as described above. The infected or transfected slides were incubated at 37 °C in a CO_2_ incubator for 48 h and stained with a PCV2-specific monoclonal antibody (Rural Technologies, Brookings, SD, USA) or a mouse polyclonal anti-*N. caninum* antibody [30] to assess the expression of the epitope tag, followed by FITC conjugated secondary antibodies as previously described [28,31]. Apple green fluorescence located in the nuclei of the PK-15 cells was indicative of PCV2 replication (Figure 2).

### 2.3. Vaccination and Challenge Study

All animal experimentation was carried out in compliance with the regulations of the Institutional Animal Care and Use Committee and Institutional Biosafety Committee of South Dakota State University (SDSU) and North Dakota State University (NDSU). Three groups of eight, 3–4-week-old conventional pigs, which were ELISA- and PCR-negative for PCV2, PRRSV, SIV, and *Mycoplasma* sp., were each administered treatments as follows: Group I—unvaccinated controls; Group II—commercial chimeric PCV1-2a inactivated vaccine (Fostera^®^ PCV MetaStim^®^, Zoetis, Inc., Kalamazoo, MI, USA), 2 mL, intramuscular; Group III—sPCV2-Vac, 10^4^TCID_50_/mL, 2 mL intranasal and 2 mL intramuscular. The vaccinated pigs were boosted on day 14 post-vaccination (DPV). The pigs were observed for any signs of reactivity to vaccination in the 28-day pre-challenge period. Two pigs in each group were randomly selected and euthanized on DPV 28 to assess vaccine safety by gross and microscopic pathological examination. All pigs were challenged with 10^4^TCID_50_ of a heterologous PCV2d virus, 2 mL intramuscular and 2 mL intranasally. Serum was collected from all pigs on day 0, DPV 14, DPV 28, and days post-challenge (DPC) 9 and 21. The experimental animals were monitored daily for signs of PCVAD, such as respiratory distress, inappetence, jaundice, anemia, and wasting. Body weights were measured on DPC 0, 9, and 21 to assess the effects of vaccination on post-challenge weight gain. The study was terminated on DPC 21, and all pigs were humanely euthanized for pathological examination.

### 2.4. Challenge Viral Replication

Post-challenge replication of the heterologous PCV2d challenge virus was detected by a PCV2d-specific qPCR, as previously described, from sera collected on DPC 0,14, and 21 [24].

### 2.5. Antibody Responses to Vaccination

Total IgG-binding antibody responses to the PCV2 capsid protein were measured by a commercial PCV2 ELISA kit (Ingezim Circovirus IgG kit, Ingenasa, Madrid, Spain). The samples were submitted to the Iowa State University Veterinary Diagnostic Laboratory and processed as per their standard operating procedures. The sample-to-positive-control (S/P) ratios generated were used for further analysis of antibody responses.

### 2.6. Virus-Neutralizing Antibody Responses

Protective virus neutralization responses were assessed by a rapid fluorescence focus neutralization (FFN) assay, as described before [25]. Sera collected at DPV 28 were tested in 4 replicates against the homologous PCV2b and heterologous PCV2a and PCV2d strains to obtain the % reduction in the number of foci due to virus neutralization when compared to the untreated virus only control.

### 2.7. Antibody Responses to the Epitope Tag

Antibody responses to the epitope tag were measured by an indirect peptide-based ELISA as described previously [24]. Samples were assessed in duplicate, and the identity of the capture antigen was verified by Western blotting prior to assay optimization, as previously described.

### 2.8. Pathological Evaluation of Tissues

Lung, liver, spleen, lymph node, ileum, kidney, and tonsil tissues from the experimental pigs at necropsy were fixed in buffered formalin for microscopic examination. Tissue sections were stained with hematoxylin and eosin and a PCV2-specific antibody for immunohistochemistry (IHC). The IHC was carried out following the standard operating procedures of the Iowa State University Veterinary Diagnostic Laboratory. Briefly, tissue sections were deparaffinized, rehydrated, and endogenous peroxidase activity was blocked with 3% hydrogen peroxide for 30 min. Tissue sections were washed in pH 7.6 0.1 M Tris-buffered saline and blocked with 150% normal goat serum for 30 min, followed by overnight incubation with a 1:250 dilution of PCV2 monoclonal antibody. Biotinylated goat anti-mouse antibody was used for detection in combination with the avidin-biotin-peroxidase (ABC) system (Pierce, IL, USA). Sections were treated with diaminobenzidine (DAB)–hydrogen peroxide, counter-stained with Harris’ hematoxylin, dehydrated, and examined microscopically. Microscopic and gross lesion scores were assigned in a blinded fashion by board-certified veterinary pathologists. Gross lung lesions were scored as the percentage of lung parenchyma involved. Enlargement of inguinal lymph nodes was assigned scores of 0–3 as follows, 0 = no enlargement, 1 = two times the normal size, 2 = three times the normal size, and 3 = four times the normal size. Viral antigen in IHC slides was scored from 1 to 4 as follows: 1 = single follicle or focus staining, 2 = rare to scattered staining, 3 = moderate staining, 4 = strong widespread staining.

### 2.9. Vaccine Viral Replication and Safety

Vaccine viral replication in the sPCV2-Vac group was quantified by a quantitative polymerase chain reaction (qPCR) assay, as previously described [24]. Briefly, a primer-probe combination specific to the foreign epitope tag was used to detect vaccine virus in the sera collected at DPV 0, 14, and 28.

The pigs administered the sPCV2-Vac and commercial vaccines were observed for any untoward clinical signs, including signs of PCVAD, swelling or reactions at the injection site, pyrexia, and neurological disorders, throughout the study. Two pigs from each group were euthanized on DPV 28 and assessed for any gross and microscopic lesions produced due to vaccination as described above.

### 2.10. Vaccine Stability

The in vitro stability of the introduced mutations was assessed by three serial passages of the rescued vaccine virus in PK-15 cells. Viral titers were assessed by the TCID_50_ method in duplicate. The capsid gene was amplified from the passaged viral culture and Sangers-sequenced to ensure the integrity of the introduced mutations. As the sPCV2 vaccine virus was not detected in the sera of vaccinated pigs at the 14-day post-vaccination time point, sequencing of viral samples obtained from pigs was not undertaken.

### 2.11. Mutation Rates under Immune Selection Pressure

To measure the relative fitness of the destabilized sPCV2 vaccine virus and the wild-type virus when subjected to immune selection pressure, the respective virus cultures were exposed to sub-neutralizing levels of PCV2b-specific antibody as previously described with some modifications [32]. Briefly, wild-type PCV2b and sPCV2-Vac virus cultures were both adjusted to 10^4^ TCID_50_/mL. Archived PCV2b-specific serum collected from infected pigs at DPI 28 [33] was diluted 1:1000 to achieve a 25% reduction in fluorescent foci compared to untreated virus control, using the rapid fluorescence focus neutralization (FFN) assay referenced above. Equal volumes (500 µL) of the diluted serum and sPCV2-Vac and wild-type PCV2b virus cultures were mixed and incubated at 37 °C for 1 h. The virus/serum mixtures were layered on pre-formed PK-15 monolayers at 50% confluence. After an incubation of 3 h at 37 °C in a CO_2_ incubator, Dulbecco’s Modified Eagle’s Medium (DMEM) containing 2% fetal bovine serum and 1× penicillin-streptomycin was added to the cells and incubated for 72 h. This process was repeated for 5 serial passages in duplicate. Three replicates at the 5th passage from each treatment were pooled and stored at −80 °C for next-generation sequencing.

### 2.12. Deep Sequencing and Bioinformatic Analysis

The collected samples were centrifuged at 5000RPM for 5 min to remove cellular debris and treated with DNAse I (Thermo Fisher Scientific, Waltham, MA, USA) according to the manufacturer’s instructions to remove non-capsid-associated DNA. Viral DNA was extracted using the QIAamp MinElute Virus kit (Qiagen, Valencia, CA, USA). The entire viral genome was amplified by PCR as previously described [13], and the DNA was purified by gel extraction. Purified DNA samples were submitted for sequencing and analysis to a commercial service provider (CD genomics, New York, NY, USA). Sequencing libraries were generated using NEBNextR Ultra™ DNA Library Prep Kit for Illumina (NEB, Ipswich, MA, USA) following the manufacturer’s recommendations. Index codes were added to attribute sequences to each sample. The DNA sample was fragmented by sonication to a size of 350 bp. The DNA fragments were end-polished, A-tailed, and ligated with the full-length adaptor for Illumina sequencing with further PCR amplification. The obtained PCR products were purified with the AMPure XP system (Beckman Coulter, Brea, CA, USA). The libraries were analyzed for size distribution by the Agilent2100 Bioanalyzer and quantified using real-time PCR, and sequenced using the Illumina PE150. The reads were curated to remove poor-quality reads and adapter sequences using the trim galore tool. Clean reads were mapped to the wild-type and sPCV2 ORF2 sequences, respectively, using the Burrow-Wheeler Aligner (bwa) toolkit. The GATK tool was used to identify single nucleotide polymorphisms (SNPs) and INDELS (insertion or deletion of bases). Only SNPs with a Qpred > 30 quality score above the threshold and with an SNP frequency of over 85% were included in assembling the consensus sequences, which were aligned with the reference sequences to annotate the detected changes. The EMBOSS cpgplot tool was used to assess dinucleotide content. In addition, DeMaSk, a substitution matrix for the prediction of impact due to single nucleotide variants (SNVs), was used to obtain the Shannon sequence entropy values and fitness scores [34]. The data obtained, excluding silent mutations, are presented in Table 1.

### 2.13. Statistical Analysis

The Minitab Version 19 software (Minitab, State College USA) or Microsoft Excel were used for analysis. The Student’s *t*-test was used to analyze ELISA and qPCR data. Non-parametric analysis was used for data that were not normally distributed, with body weights and lesion scores being analyzed by the Mann–Whitney U-test. A cut-off value of *p* < 0.05 was assigned for significance levels for all statistical tests, as depicted in the figures, in addition to the mean and standard deviations.

## 3. Results

### 3.1. Codon Modification Does Not Compromise Viral Rescue and Replication

Mutation of 22 selected serine and leucine codons in the PCV2b capsid protein in the backbone of a PCV2b infectious clone (Figure 1, Table 1) did not affect the laboratory culture and replication of the sPCV2-Vac construct. Recoding serine and leucine codons in the ORF2 of sPCV2-Vac did not significantly change the overall CpG/UpA dinucleotide content as the observed/expected ratio for both the wild-type and recoded ORF2 sequences remained >0.60. The GC content was also similar at 51.14% and 48.86% for the wild-type and mutated sequences, respectively. The overall genetic similarity between the wild-type PCV2b and sPCV2-Vac was not significantly altered by the recoding of the serine and leucine codons. The ORF2 and the entire genome were 96% and 98% similar for sPCV2-Vac and the wild-type virus, respectively. Transfection of PK-15 cells resulted in the production of a viable virus confirmed by the detection of an intranuclear immunofluorescent signal (Figure 2). Expression of the foreign epitope tag was also detected by an immunofluorescence-based assay. The tag that consisted of an immunogenic peptide from the SRS2 protein of an Apicomplexan parasite, *Neospora caninum*, was detected using a polyclonal *N. caninum*-specific antibody as previously reported (data not shown) [24]. The recombinant virus culture remained infective for three serial passages tested in PK-15 cells. The mean viral titers did not differ significantly between sPCV2-Vac (10^4.6^ TCID_50_/mL) and the wild-type virus (10^4.75^ TCID_50_/mL) in the passages tested. Sangers’ sequencing of the sPCV2-Vac construct did not reveal any changes to the modified codons, indicating that the mutations introduced were stable under standard virus culture conditions.

### 3.2. Vaccination of Pigs with sPCV2-Vac Protects against Replication of the Heterologous Challenge Virus

**Figure 3:**
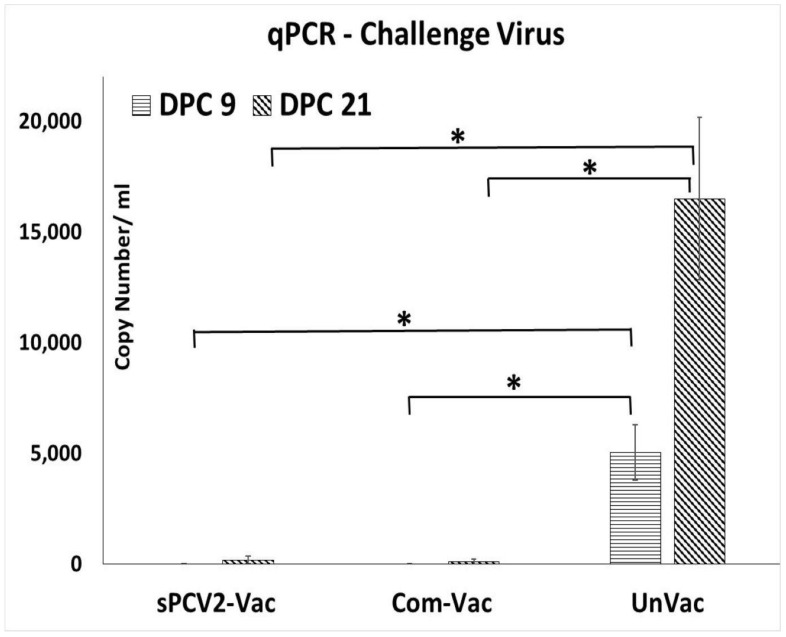
Protection against heterologous challenge viral replication: Challenge viral replication as measured by a PCV2d-specific qPCR. *X*-axis—treatment groups—horizontal lines—days post-challenge 9, slanted lines—days post-challenge 21, *Y*-axis—copy numbers/mL serum. Error bars represent the standard error of the mean. * Significantly different from the unvaccinated control group by the Student *t*-test at *p* ≤ 0.05 (horizontal bar).

Prior to the study, all pigs were screened and confirmed to be PCV2-negative. The vaccine virus was not detected by qPCR at 14- or 28-days post-vaccination (DPV) in the pigs administered sPCV2-Vac, indicating that the codon destabilized PCV2 virus was attenuated in pigs. Following challenge with a heterologous PCV2d virus, viral replication in the unvaccinated pigs followed a pattern typical for PCV2 infection, with virus titers showing an increasing trend during the 21-day post-challenge period. In contrast, the PCV2 challenge virus was not detected in pigs administered the sPCV2-Vac or the commercial control vaccine at DPC 9. At DPC 21, one pig in the sPCV2-Vac group (3.01 ± 2.62 mean log copy numbers/mL serum) and two pigs in the commercial vaccine group (1.92 ± 2.24 mean log copy numbers/mL serum) were very low PCR positives (Figure 3). There was no significant difference between the vaccine groups, and the live virus could not be isolated from the pig sera.

### 3.3. Vaccination with sPCV2-Vac Elicits Low-Level IgG Responses

**Figure 4:**
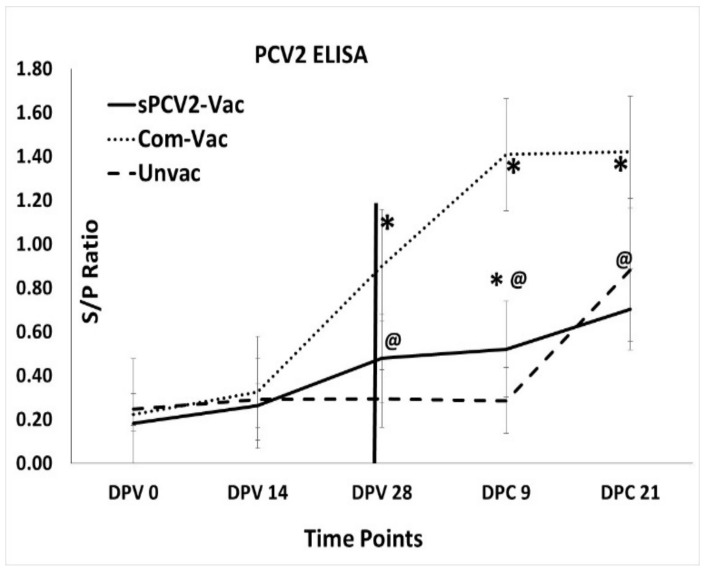
Anti-PCV2 IgG responses. Mean binding antibody responses to PCV2 as measured by a PCV2-specific commercial ELISA kit. *X*-axis—time points at which serum was collected, DPV—days post-vaccination, DPC—days post-challenge. Error bars represent the standard deviation. *Y*-axis—signal to positive (S/P) ratio, dots—commercial vaccine, solid black—sPCV2-Vac, dashes—unvaccinated pigs. * Significantly different from the unvaccinated control group by the Student’s *t*-test at *p* ≤ 0.05, @ significantly different from the commercial vaccine control group by the Student’s *t*-test at *p* ≤ 0.05.

Assessment of PCV2-specific binding IgG antibody responses using a commercial ELISA showed that the commercial vaccine elicited robust responses, while the unvaccinated pigs remained serologically negative until challenge. However, antibody responses in the sPCV2-Vac group showed no significant differences compared to the unvaccinated controls until challenge, after which an incremental trend was observed. The antibody responses of the sPCV2-Vac group were significantly lower than the commercial control group at DPV 28 and thereafter, while they were significantly different from the unvaccinated group only at DPC 9. Antibody responses in the unvaccinated group continued to increase during the 21-day post-challenge period (Figure 4).

### 3.4. Vaccination Elicits Antibody Responses to the Foreign Epitope Tag

**Figure 5:**
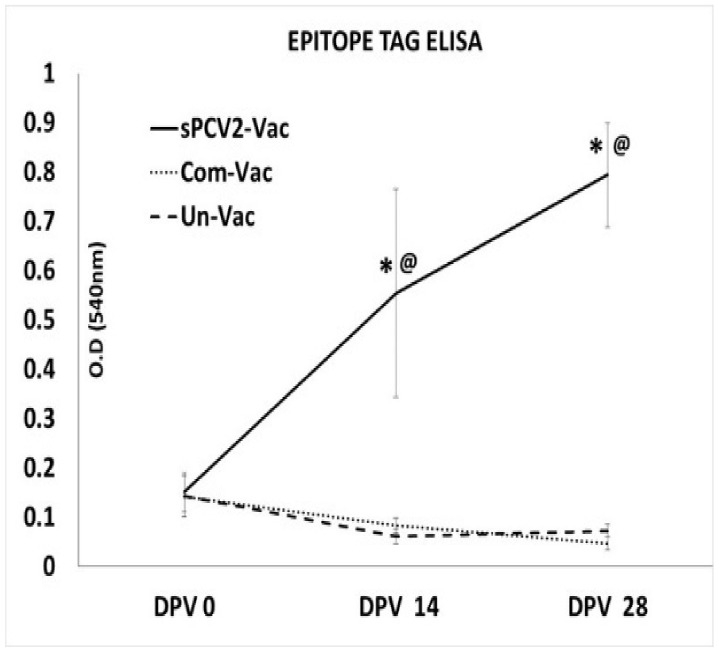
Antibody responses to the SRS2 epitope tag. Mean optical density values of sera obtained by an SRS2 epitope tag-specific ELISA. *X*-axis—time points at which serum was collected, DPV—days post-vaccination. Error bars represent the standard deviation, *Y*-axis—optical density (OD) values, dots—commercial vaccine, solid black—sPCV2-Vac, dashes—unvaccinated pigs. * Significantly different from the unvaccinated control group by the Student’s *t*-test at *p* ≤ 0.05, @ Significantly different from the commercial vaccine control group by the Student’s *t*-test at *p* ≤ 0.05.

As expected, vaccination with the sPCV2-Vac elicited SRS2 tag-specific antibody responses in the sPCV2-Vac group but not in the commercial vaccine group or unvaccinated group (Figure 5).

### 3.5. Vaccination with sPCV2-Vac Elicits Robust, Cross-Protective Virus-Neutralizing Antibody Responses

**Figure 6:**
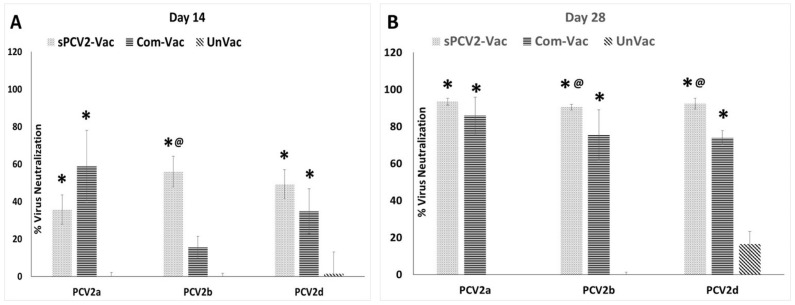
Virus neutralization responses in vaccinated pigs: Mean percentage virus neutralization as measured by a rapid fluorescent focus inhibition test at (**A**) 14 days post-vaccination (**B**) 28 days post-vaccination measured against a heterologous PCV2a strain, homologous PCV2b strain, and heterologous PCV2d strain. *X*-axis—experimental groups, *Y*-axis—percentage virus neutralization, dots—sPCV2-Vac, horizontal lines—commercial vaccine, slanted lines—unvaccinated group, * significantly different from the unvaccinated control group by the Student’s *t*-test at *p* ≤ 0.05, @ significantly different from the commercial vaccine control group by the Student’s *t*-test at *p* ≤ 0.05. Error bars represent the standard deviation.

Although binding antibody responses elicited by vaccination with sPCV2-Vac were low, strong virus-neutralizing antibody responses were observed not only against the homologous PCV2b subtype but also against the heterologous PCV2a and PCV2d subtypes. The differences were significantly different from the commercial vaccine control for PCV2b at both DPV 14 and 28 and for PCV2d at DPV21 (Figure 6A,B). The commercial vaccine, which contains the PCV2a subtype, had the lowest neutralizing activity against the PCV2b subtype while neutralizing activity against the homologous PCV2a subtype was robust but not significantly different from sPCV2-Vac. As expected, serum from the unvaccinated pigs did not neutralize the three PCV2 subtypes (Figure 6).

### 3.6. Vaccinated Pigs Are Protected against the Development of Lesions Due to PCV2d Challenge

**Figure 7:**
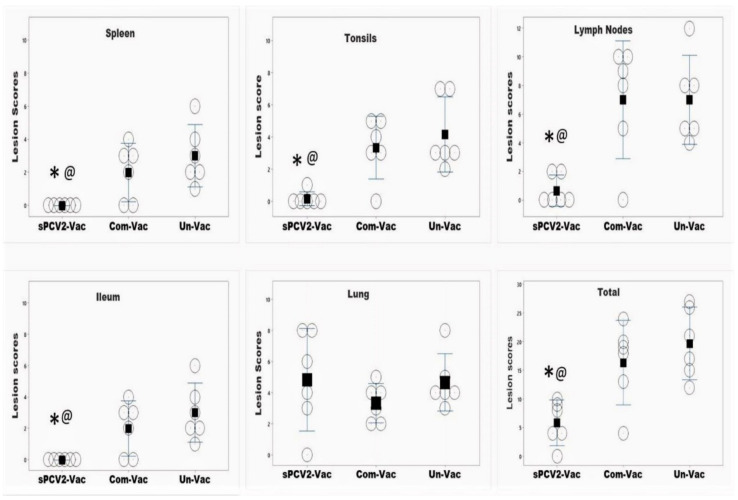
Lesion scores in the major organs: The lesion scores for the major organs recorded during necropsy at 21 days post-challenge are depicted as the sum of the gross, microscopic, and immunohistochemistry scores for each tissue. *X*-axis—group, *Y*-axis—score, bars—95% confidence interval of the means, open circles—individual values, solid square—mean, * significantly different from the unvaccinated control group and, @ significantly different from the commercial vaccine control group by the Mann–Whitney U-test at *p* ≤ 0.05.

Lymph nodes, spleen, and tonsils, which are the sites of predilection for PCV2, had low to no detectable lesions in pigs vaccinated with sPCV2-Vac. In contrast, the mean lesion scores in these tissues for the pigs vaccinated with the commercial vaccine were comparable to or higher than the unvaccinated group. Lesions were not detected in the ileum for pigs in the sPCV2-Vac group, while mild intestinal lesions were detected in both control groups. The lung lesion scores for the sPCV2-Vac group were higher than both control groups, but the difference was not statistically significant. The total lesion scores of the sPCV2-Vac group were significantly lower than both the control groups, while it was not significantly different from the unvaccinated control group for the pigs administered the commercial vaccine (Figure 7).

### 3.7. Vaccinated Pigs Are Protected against Weight Loss

**Figure 8:**
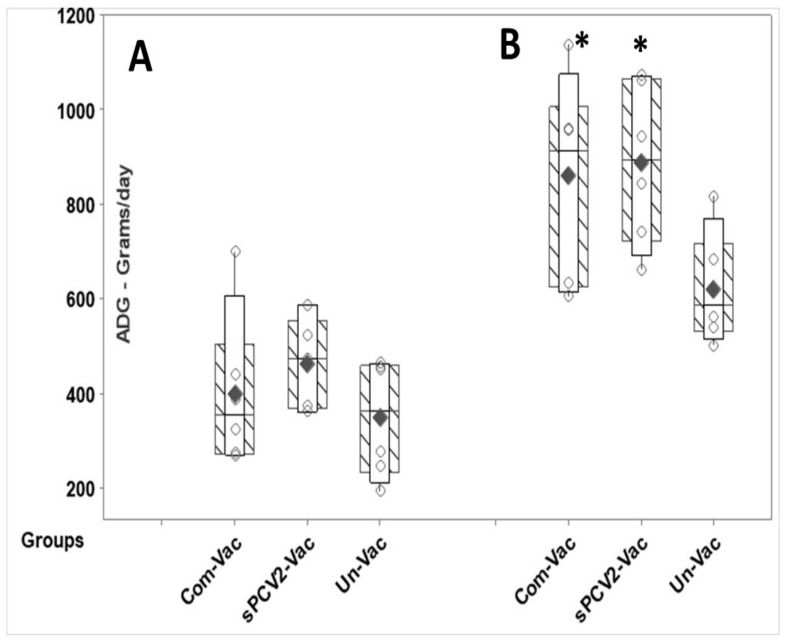
Weight gain in vaccinated pigs: Protection against weight loss due to challenge with the heterologous PCV2d virus is represented as the average daily weight gain (ADG). *X*-axis—experimental groups, *Y*-axis—ADG in grams per day, **A**—10 days post-challenge **B**—21 days post-challenge. Boxes with slanted lines—interquartile range, whiskers—range, unfilled vertical bar—95% confidence interval, horizontal bar—median, solid black diamonds—mean, circles—individual values. * Significantly different from the unvaccinated control group by the Student’s *t*-test at *p* ≤ 0.05. No significant differences were observed between the commercial vaccine group and test vaccine group at both time points tested.

A comparison of post-challenge weight gain between the vaccinated and unvaccinated pigs showed that pigs in the sPCV2-Vac gained significantly more weight than the unvaccinated controls at DPC 21, while the differences were not significant at DPC 10. Weight gain in the commercial vaccine group and the sPCV2-Vac group were similar at both time points evaluated, with no significant differences between the groups (Figure 8). Overt signs of PCV2 infection were not observed in any of the evaluated groups in the post-challenge period, as is common in experimental PCV2 infections.

### 3.8. sPCV2-Vac Is Safe

The sPCV2-Vac virus was not detected in the serum of pigs at DPV 14 or at DPV 28 by qPCR. Hence sequencing of sPCV2-Vac to determine whether the in vivo vaccine viral clearance was due to the accumulation of stop codons could not be undertaken. Clinical signs of PCVAD were not observed during the pre-challenge period in pigs administered sPCV2-Vac. No significant lesions were observed in the major organs of the two pigs euthanized from each group prior to challenge to assess vaccine safety, indicating that vaccination with the sPCV2-Vac did not cause untoward reactions.

### 3.9. sPCV2-Vac Subjected to In Vitro Immune Selection Pressure Accumulates Several Stop Mutations

Deep sequencing of the sPCV2-Vac and wild-type PCV2b viruses subjected to immune selection pressure passaging in the presence of sub-neutralizing antibodies resulted in a total of 1711 MB of raw reads and 0.97 GB of clean reads. A total of 28 single nucleotide variants (SNVs) were detected for sPCV2-Vac, of which 7 SNVs resulted in silent mutations (not included in Table 1). The expected conversion of serine and leucine codons to stop codons was detected in 5 out of the 22 recoded sites. All SNVs for the sPCV2-Vac treatment were located within the first 90 N terminal amino acids. During serial passage with sub-neutralizing antibodies, the titers of the wild-type virus reduced by about one log while viral replication diminished significantly over the passages for sPCV2-Vac, and passaging was discontinued. In contrast, changes in the wild-type PCV2b passaged under the same immune selection pressure conditions resulted in just one single amino acid mutation of leucine to valine (L 167 V) (Table 1, S1-Supplemental data file) and other two silent mutations.

Besides the expected stop codons, non-synonymous changes were detected in three of the recoded codons. In addition, eight non-synonymous changes were detected in nontarget codons, which either were located adjacent to the target codons or were one codon removed. The DeMaSk tool [34] was used to assess the effect of the nontarget mutations (Table 1) on viral fitness. The Shannon’s sequence entropy scores (25), which predict the naturally occurring frequency of substitution for a given amino acid across homologs, were high for eight nontarget substitutions. Hence, they were unlikely to have a significant structural or functional effect (Table 1). However, seven other nontarget substitutions had negative fitness scores ranging from 0 to −0.4, on a scale where 0 equates to no change in fitness, and increasing negative scores from 0 to −1.0 predict an increasing loss of fitness [34]. The DeMaSk fitness score is a composite of Shannon’s sequence entropy, frequency of the single nucleotide variant in homologs, and the directional amino acid substitution matrix generated by deep mutational scanning data [34].

## 4. Discussion

Currently, classified CRESS viruses with pathogenic potential and economic impact in farm animals are limited to circoviruses and gyroviruses. However, the explosion in the discovery of new CRESS viruses, their vast genetic diversity, and their ability to adapt to a wide host range [17,35] warrant the investigation into improved platforms for their prevention. Unlike sPCV2-Vac, the commercial PCV2 product used in the study is extensively standardized and dose optimized. Hence, direct comparisons to the experimental sPCV2-Vac are avoided. The rapid clearance of sPCV2-Vac in vaccinated pigs likely resulted in the low magnitude of the pre-challenge IgG response (Figure 4). However, consistent with our previous findings for minimally replicative vaccines [3], the protective virus-neutralizing antibody response was not affected (Figure 6). In fact, compared to the controls, virus neutralization was broader and more robust against the currently circulating heterologous PCV2d strain, likely contributing to the effective early clearance of the heterologous PCV2d challenge virus (Figure 3), protection against lymphoid lesions (Figure 7), and improved post-challenge weight gain (Figure 8). While exploring the mechanisms by which low rather than high levels of exposure to protective antigens elicits superior immunity is beyond the scope of this study, it is well established that the effective initial priming of the immune response is critical and strongly influenced by viral proteins and nucleic acids generated from the first round of viral replication. Further, subunit and inactivated vaccines are depleted of viral DNA, which can potentially trigger effective innate immunity. The reduced competition for MHC-I or MHC-II presentation from irrelevant viral antigens in single-cycle vaccine vectors was shown to enhance responses to the critical antigens [36]. Similarly, lower doses of peptide antigen, rather than high doses, preferentially primed high avidity CD4^+^T cells for cancer immunotherapy, stimulating both antibody responses and cytotoxic T cell responses, instead of skewing the response toward one arm of the immune system [37].

While a limitation of this study is that cell-mediated immune responses were not studied, immune responses to T cell epitopes conserved between subtypes could have also played a role in improving heterologous protection. Although the low-level detection of viral DNA in three pigs at DPC 21 (Figure 3) cannot be equated to live viral replication in the absence of virus isolation, it is likely that with further dose optimization, the sPCV2-Vac can achieve sterilizing immunity. As the serum is a commonly accepted matrix for PCV2 detection by PCR [38], and wild-type PCV2 replicates to high titers for 3–4 weeks post-infection in experimental models (Figure 3), tissues, nasal or fecal swabs, or earlier time points were not sampled respectively. Therefore, the virus could not be sequenced from in vivo samples to detect stop mutations. However, compared to the wild-type PCV2, which replicated to high titers (Figure 3) and produced lesions in lymphoid organs (Figure 7), the sPCV2-Vac virus was clearly attenuated in vivo and safe as it was not detectable by qPCR in the serum of vaccinated pigs by DPV 14. The presence of the marker peptide did not negatively influence vaccine-mediated immunity as significant antibody responses to both the marker (Figure 5) and the PCV2 capsid protein (Figure 4) were detected, and sPCV2-Vac was highly effective and safe. Conversely, mutation of the serine and leucine codons did not affect the stability of the epitope tag.

The observations that the mutation rates of ssDNA viruses are comparable to some RNA viruses, and are significantly higher than their eukaryotic hosts or double-stranded DNA viruses, are well documented. While the high mutation rates of RNA viruses are readily explained by the poor proof-reading ability of RNA polymerases, very little is known regarding the mechanisms of mutation in ssDNA viruses [39]. Multiple factors such as short replication and lysis times, genome size, single-strandedness, methylation status [16,40], the processivity or fidelity of traditional helicases, polymerases, and DNA-binding proteins on ssDNA [22,23] or mutagenic reactive oxygen species (ROS) released in virally infected cells [41,42] are suggested influencers of viral mutation rates [39]. The high mutation rates of the single-stranded plant Gemini viruses were recently attributed to their dependence on atypical, low fidelity, translesion synthesis (TLS) polymerases for replication [43]. Similarly, Epstein–Barr virus uses TLS polymerase Pol η for the repair of viral DNA [44]. Low fidelity, TLS polymerases participate in the cellular DNA damage response by inserting nucleotides with low fidelity in DNA breaks so that DNA replication can continue to ensure cell survival [45]. The rolling circle replication of PCV2 involving helicase-mediated unwinding, nicking, template swapping, formation, and resolution of intermediate double-stranded DNA concatemers creates several opportunities for viral DNA excision and repair [6]. While experimental evidence is lacking, it is possible that the DNA damage response and repair mechanisms for PCV2 may involve TLS polymerases [16], with the additional mutational potential generated by the destabilization of the serine and leucine codons in sPCV2-Vac (Table 1), resulting in rapid attenuation.

Similarly, the significantly accelerated rate of mutation of sPCV2-Vac under immune selection pressure could be hypothetically explained by recent discoveries regarding post-cell entry virus neutralization mechanisms, which demonstrate that naked viruses can be directly endocytosed as complexes bound to antibodies. A cytosolic restriction factor, TRIM21, tags the antigen antibody complexes for ubiquitination by interacting with the Fc regions [46]. Interestingly, TRIM21 also regulates the cellular redox state, as cells deficient in TRIM21 are protected from oxidative damage [47]. Both TRIM21 [48] and ROS, which can host and viral DNA damage [49], are several folds upregulated in PK-15 cells following PCV2 infection. The intersecting roles of TRIM21 and the oxidative damage pathways in virus neutralization mechanisms and cellular vial pathogenesis, combined with the low fidelity activity of atypical TLS polymerases, could hypothetically increase mutation rates in the codon destabilized sPCV2-Vac substantially when subjected to sub-neutralizing antibody pressure. Although this possible explanation needs further experimentation, it is plausible given the experimental evidence for TLS polymerase usage in other DNA viruses, post-entry virus neutralization mechanisms, and data on oxidative damage and upregulation of TRIM21 in PCV2 infected cells.

Although the changes detected in amino acids 57–83 (Table 1) are located in a major immunodominant region of the PCV2 capsid protein with residue 59 being critical for the recognition of PCV2 by antibodies [24,50], the mutations observed are more likely due to the contextual effects of the recoded serine and leucine codons than true immune selection. Moura et al. recently showed that apparently neutral synonymous mutations are actually constrained by the need to maintain their codon context or codon pair usage at the DNA level, and changes to the third nucleotide of the preceding codon had a strong modulatory effect on the adjacent codon [51]. The occurrence of mutations in nontarget codons was not described for serine, and leucine codon destabilized influenza and coxsackie viruses subjected to mutagenic pressure [21]. Regardless, stop codons were detected in 100% of the sequences by deep sequencing (Table 1). Therefore, the survival of any variants adapted to pre-existing immunity is highly unlikely under field conditions where pre-existing immunity to PCV2 is common. An increase in CpG/UpA dinucleotide content in viral genomes often accompanies codon or codon pair deoptimization approaches, resulting in recognition as non-self in hosts with low CpG/UpA content genomes, contributing to the attenuation of codon deoptimized vaccines by recognition [52]. This mechanism of attenuation is unlikely for sPCV2-Vac as there was no overall change in the CpG/UpA dinucleotide content. The preferred codons for leucine and serine in PCV2 resemble the porcine host and are CTC and TGC/TCC, respectively [53]. While the mutations introduced in this study did not target the retention of the preferred codon usage and favored TTG codons for leucine, the stability of sPCV2-Vac was not affected under normal in vitro culture conditions.

Thus, we demonstrate for the first time that mutation of serine and leucine codons of the PCV2 capsid to target suicidal replication of the vaccine construct is a feasible strategy for improving the safety and heterosubtypic protection of attenuated PCV2 vaccines and can be potentially applied to other single-stranded DNA viruses. The increased susceptibility for self-limiting replication of the sPCV2-Vac under sub-neutralizing antibody selection pressure could be an added safety advantage under field conditions when pre-existing immunity to PCV2 is common. Given their wide host range and the recent increases in metagenomic detection of new porcine circoviruses and other CRESS DNA viruses, the availability of improved vaccine development approaches with wide applicability to viruses with high mutation rates have potential positive benefits for animal and human health.

## 5. Patents

The submitted work is protected by a provisional patent application, in compliance with the regulations of North Dakota State University.

## Figures and Tables

**Table 1:** Mutations under immune selection pressure.

Initial Codon	Codon Change	Mutation/Position	Frequency of Detection	Type	Entropy	Fitness
sPCV2-Vac						
TTA	TGA	L 23 STOP *	1.0	Tv		
CGC	GGC	R 24 G	1.0	Tv	0.54	−0.3
TTG	TGG	L 29 W	1.0	Tv	0.18	−0.39
TAC	GAC	Y 36 D	0.27	Tv	0.18	−0.39
TGG	AGG	W 38 R	0.26	Tv	0.16	−0.30
TTA	TGA	L 49 STOP *	0.18	Tv		
TCA	GCA	S 50 G	0.99	Tv	0.99	−0.18
TCA	TGA	S 50 STOP *	1.0	Tv		
ATC	ACC	I 57 T	0.18	Ti	1.07	−0.27
CGA	CCA	R 59 P	0.18	Tv	2.1	0.34
TCG	TGG	S 66 W	1.0	Tv	0.47	−0.31
AAT	ACT	N 77 T	0.09	Tv	1.74	−0.11
TTG	TAG	L 80 Stop *	1.0	Tv		
CCC	GCC	P 81 A	0.91	Tv	0.35	−0.28
GGA	CGA	G 83 R	0.08	Tv	0.47	−0.40
TCA	TAA	S 90 Stop *	1.0	Tv		
PCV2b Wild-type						
CTA	GTA	L 167 V	0.99	Tv	0.69	−0.25

Shaded text—target recoded serine and leucine codons; * recoded serine or leucine codons that mutated to stop codons. Ti—transition, Tv—transversion; entropy score—Shannon’s sequence entropy score from DeMaSk. Higher values indicate residues with a greater tendency toward substitution. Low values indicate conserved residues with high fitness consequences when substituted; fitness score—0—no fitness change, <0—loss of fitness, >0—gain of fitness.

## Data Availability

The data presented in this study are available on request.

## Supplementary Materials

The following are available online at https://www.mdpi.com/article/10.3390/v13091676/s1. Supplementary file S1. File containing DNA sequences of the wild-type PCV2b and sPCV2-Vac.
