# Supplementary material for: Harnessing the Genetic Plasticity of Porcine Circovirus Type 2 to Target Suicidal Replication
Source: Viruses. 2021 Aug 24;13(9):1676. doi: 10.3390/v13091676 (PMC8473201; doi:10.3390/v13091676)
Supplement: Supplementary file 1 [file viruses-13-01676-s001.zip › viruses-1340331-supplementary.pdf]

Sequences - Supplementary Data S1

Wildtype PCV2b 41513 ORF2 DNA

ATGACGTATCCAAGGAGGCGTTACCGGAGAAGAAGACACCGCCCCCGCAGCCATCTTGGCCAGATCC
TCCGCCGCCGCCCCTGGCTCGTCCACCCCCGCCACCGTTACCGCTGGAGAAGGAAAAATGGCATCTTC
AACACCCGCCTATCCCGCACCTTCGGATATACTATCAAGCGAACCACAGTCAGAACGCCCTCCTGGGC
GGTGGACATGATGAGATTCAATATTAATGCCTTTCTTCCCCCAGGAGGGGGCTCAAACCCCCGCTCTG
TGCCCTTTGAATACTACAGAATAAGAAAGGTTAAGGTTGAATTCTGGCCCTGCTCCCCGATCACCCAG
GGTGACAGGGGAGTGGGCTCCAGTGCTGTTATTCTAGATGATAACTTTGTAACAAAGGCCACAGCCCT
CACCTATGACCCCTATGTAAACTACTCCTCCCGCCATACCATAACCCAGCCCTTCTCCTACCACTCCCG
CTACTTTACCCCCAAACCTGTACTAGATTCCACTATTGATTACTTCCAACCAAACAACAAAAGAAACC
AGCTGTGGCTGAGACTACAAACTGCTGGAAATGTAGACCACGTAGGCCTCGGCACTGCGTTCGAAAA
CAGTATATACGACCAGGGATACAATATCCGTGTAACCATGTATGTACAATTCAGAGAATTTAATCTTA
AAGACCCCCCACTTAACCCTTAA

sPCV2-Vac ORF2 DNA with mutated serine and leucine codons in small letters

ATGACGTATCCAAGGAGGCGTTACCGGAGAAGAAGACACCGCCCCCGCAGCCATCTTGGCCAGATCtt
aCGCCGCCGCCCCTGGttgGTCCACCCCCGCCACCGTTACCGCTGGAGAAGGAAAAATGGCATCTTCAA
CACCCGCttatcaCGCACCTTCGGATATACTATCAAGCGAACCACAGTCAGAACGCCCtcgTGGGCGGTGG
ACATGATGAGATTCAATATTAATGCCTTTttgCCCCCAGGAGGGGGCTCAAACCCCCGCtcaGTGCCCTTT
GAATACTACAGAATAAGAAAGGTTAAGGTTGAATTCTGGCCCTGCtcaCCGATCACCCAGGGTGACAG
GGGAGTGGGCtcgtcaGCTGTTATTttgGATGATAACTTTGTAACAAAGGCCACAGCCttaACCTATGACCCC
TATGTAAACTACtcatcgCGCCATACCATAACCCAGCCCTTCtcgTACCACtcaCGCTACTTTACCCCCAAAC
CTGTAttaGATtcaACTATTGATTACTTCCAACCAAACAACAAAAGAAACCAGttgTGGttgAGAttaCAAACT
GCTGGAAATGTAGACCACGTAGGCttgGGCACTGCGTTCGAAAACAGTATATACGACCAGGGATACAA
TATCCGTGTAACCATGTATGTACAATTCAGAGAATTTAATCTTAAAGACCCCCCACTTAACCCTTAA

PCV2b 41513 ORF2 protein – wildtype and mutated sequence

MTYPRRRYRRRRHRPRSHLGQILRRRPWLVHPRHRYRWRRKNGIFNTRLSRTFGYTIKRTTVRTPSWAVDM
MRFNINAFLPPGGGSNPRSVPFEYYRIRKVKVEFWPCSPITQGDRGVGSSAVILDDNFVTKATALTYDPYVNY
SSRHTITQPFSYHSRYFTPKPVLDSTIDYFQPNNKRNQLWLRLQTAGNVDHVGLGTAFENSIYDQGYNIRVT
MYVQFREFNLKDPPLNP

Sequencing of samples passaged under selection pressure

S PCV2-Vac DNA sequence

ATGACGTATCCAAGGAGGCGTTACCGGAGAAGAAGACACCGCCCCCGCAGCCATCTTGGCCAGATCt
GagGCCGCCGCCC
CTGGtggGTCCACCCCCGCCACCGTgACCGCaGGAGAAGGAAAAATGGCATCTTCAACACCCGCtGatGa
CGCACCTTCG
GATATACTAcCAAGCcgACCACAGTCAGAACGCCCtGgTGGGCGGTGGACATGATGAGATTCAATATTA
cTGCtTTTtAg
gCCCCAcGAGGGGGCTCgAACCCCCGCtAaGTGCCCTTTGAATACTACAGAATAAGAAAGGTTAAGGTT
GAATTCTGGCC
CTGCtcaCCGATCACCCAGGGTGACAGGGGAGTGGGCtcgtcaGCTGTTATTttgGATGATAACTTTGTAACA
AAGGCCA

CAGCCttaACCTATGACCCCTATGTAAACTACtcatcgCGCCATACCATAACCCAGCCCTTCtcgTACCACtca
CGCTAC
TTTACCCCCAAACCTGTAttaGATtcaACTATTGATTACTTCCAACCAAACAACAAAAGAAACCAGttgTGG
ttgAGAtt
aCAAACTGCTGGAAATGTAGACCACGTAGGCttgGGCACTGCGTTCGAAAACAGTATATACGACCAGG
GATACAATATCC
GTGTAACCATGTATGTACAATTCAGAGAATTTAATCTTAAAGACCCCCCACTTAACCCTTAA_

S PCV2-Vac Protein sequence

MTYPRRRYRRRRHRPRSHLGQI.GRRPWWVHPRHRDRRRRKNGIFNTR..RTFGYTTKPTTVRTPWWAVDM
MRFNITAF.
APRGGSNPR.VPFEYYRIRKVKVEFWPCSPITQGDRGVGSSAVILDDNFVTKATALTYDPYVNYSSRHTITQPFS
YHSRY
FTPKPVLDSTIDYFQPNNKRNQLWLRLQTAGNVDHVGLGTAFENSIYDQGYNIRVTMYVQFREFNLKDPPL
NP_

_

Wildtype NGS sequencing

Wildtype : Only 3 mutations, two silent, and 1 Leu to Val were detected.

Wildtype PCV2b 41513 ORF2 DNA sequence

ATGACGTATCCAAGGAGGCGTTACCGGAGAAGAAGACACCGCCCCCGCAGCCATCTTGGCCAGATCC
TCCGCCGCCGCCC
CTGGCTCGTCCACCCCCGCCACCGTTACCGCTGGAGAAGGAAAAATGGCATCTTCAACACCCGCCTAT
CCCGCACCTTCG
GATATACTATCAAGCGAACCACAGTCAGAACGCCCTCCTGGGCGGTGGACATGATGAGATTCAATATT
AATGCTTTTCTT
CCCCCAGGAGGGGGCTCAAACCCCCGCTCTGTGCCCTTTGAATACTACAGAATAAGAAAGGTTAAGG
TTGAATTCTGGCC
CTGCTCCCCGATCACCCAGGGTGACAGGGGAGTGGGCTCCAGTGCTGTTATTCTAGATGATAACTTTGT
AACAAAGGCCA
CAGCCCTCACCTATGACCCCTATGTAAACTACTCCTCCCGCCATACCATAACCCAGCCCTTCTCCTACC
ACTCCCGCTAC
TTTACCCCCAAACCTGTAGTAGATTCCACTATTGATTACTTCCAACCAAACAACAAAAGAAACCAGCT
GTGGCTGAGACT
ACAAACTGCTGGAAATGTAGACCACGTAGGCCTCGGCACTGCGTTCGAAAACAGTATATACGACCAG
GGTTACAATATCC
GTGTAACCATGTATGTACAATTCAGAGAATTTAATCTTAAAGACCCCCCACTTAACCCTTAA_

Wildtype PCV2b 41513 ORF2 protein sequence

MTYPRRRYRRRRHRPRSHLGQILRRRPWLVHPRHRYRWRRKNGIFNTRLSRTFGYTIKRTTVRTPSWAVDM
MRFNINAFL
PPGGGSNPRSVPFEYYRIRKVKVEFWPCSPITQGDRGVGSSAVILDDNFVTKATALTYDPYVNYSSRHTITQPF
SYHSRY

FTPKPVVDSTIDYFQPNNKRNQLWLRLQTAGNVDHVGLGTAFENSIYDQGYNIRVTMYVQFREFNLKDPPL
NP_
